# Myosins 1 and 6, myosin light chain kinase, actin and microtubules cooperate during antibody-mediated internalisation and trafficking of membrane-expressed viral antigens in feline infectious peritonitis virus infected monocytes

**DOI:** 10.1186/1297-9716-45-17

**Published:** 2014-02-12

**Authors:** Hannah L Dewerchin, Lowiese M Desmarets, Ytse Noppe, Hans J Nauwynck

**Affiliations:** 1 Department of Virology, Parasitology and Immunology, Faculty of Veterinary Medicine, Ghent University, Salisburylaan 133, 9820 Merelbeke, Belgium

## Abstract

Monocytes infected with feline infectious peritonitis virus, a coronavirus, express viral proteins in their plasma membranes. Upon binding of antibodies, these proteins are quickly internalised through a new clathrin- and caveolae-independent internalisation pathway. By doing so, the infected monocytes can escape antibody-dependent cell lysis. In the present study, we investigated which kinases and cytoskeletal proteins are of importance during internalisation and subsequent intracellular transport. The experiments showed that myosin light chain kinase (MLCK) and myosin 1 are crucial for the initiation of the internalisation. With co-localisation stainings, it was found that MLCK and myosin 1 co-localise with antigens even before internalisation started. Myosin 6 co-localised with the internalising complexes during passage through the cortical actin, were it might play a role in moving or disintegrating actin filaments, to overcome the actin barrier. One minute after internalisation started, vesicles had passed the cortical actin, co-localised with microtubules and association with myosin 6 was lost. The vesicles were further transported over the microtubules and accumulated at the microtubule organising centre after 10 to 30 min. Intracellular trafficking over microtubules was mediated by MLCK, myosin 1 and a small actin tail. Since inhibiting MLCK with ML-7 was so efficient in blocking the internalisation pathway, this target can be used for the development of a new treatment for FIPV.

## Introduction

Two genetically highly similar biotypes of coronaviruses are described in cats: feline infectious peritonitis virus (FIPV) and feline enteric coronavirus (FECV). These coronaviruses can infect both cats and other members of the Felidae family. An infection with FECV is usually sub-clinical, except in young kittens where it may cause mild to severe diarrhoea [1]. In contrast, FIPV infection causes a chronic and very often fatal pleuritis/peritonitis. In fact, it is the most important cause of death of infectious origin in cats. Cats with clinical FIP often have very high titers of FIPV-specific antibodies. Yet, these antibodies are not able to block infection, which suggests that antibodies and antibody-driven immune effectors are not able to efficiently clear the body from virus and/or virus-infected cells.

In previous work, we presented some immune evasion strategies used by FIPV that could clarify why antibodies seem to be unable to identify infected cells and/or mark them for antibody-dependent cell lysis. We found that only half of the infected monocytes express viral proteins on their surface [2]. In the cells that do express viral proteins, these proteins are internalised upon antibody addition through a highly efficient and fast process resulting in FIPV-infected cells without visually detectable viral proteins on their plasma membrane [3]. The fact that no viral antigens can be found on FIPV infected monocytes isolated from naturally infected FIP cats while this expression returns after in vitro cultivation, is a strong indication that this immune evasion strategy occurs in vivo [4]. We then went on to elucidate through which internalisation pathway these antigen-antibody complexes are internalised.

Ligands can be internalised into cells via several pathways. There are 4 “classical” pathways: phagocytosis, macropinocytosis, clathrin-mediated internalisation and caveolae-mediated internalisation (for extensive reviews readers are referred to [5-11]) and 5 less well defined “non-classical” pathways. These latter pathways are distinguished from one another by their dependence on rafts, dynamin and Rho-GTPases. Two pathways are dependent on dynamin. A first pathway is used by the interleukin 2 (Il2) receptor for uptake of Il2 in leukocytes and is dependent on rafts and (an) unidentified Rho-GTPase(s) [12]. This pathway might also be used by cellular prion proteins [13]. A second dynamin-dependent non-classical pathway is actin and Rho-kinase dependent but independent of rafts and is used by intracellular adhesion molecule-1 and platelet-endothelial cell adhesion molecule-1 [14]. Of the 3 dynamin-independent pathways, 1 is dependent on rafts and Cdc42 (a Rho-GTPase) and is utilised by GPI-anchored proteins; like the folate receptor [15,16]. Another dynamin-independent pathway is used by Menkes disease ATPase (ATP7a), a defective copper transporting ATPase and is also independent from rafts but is regulated by Rac1 (a Rho-GTPase) [17]. The third dynamin-independent internalisation pathway was presented in our previous work and is the pathway through which viral surface expressed proteins in FIPV infected monocytes are internalised. This pathway, the fifth non-classical pathway, occurs independently from rafts, dynamin and rho-GTPases [18]. Surely more pathways await their discovery.

Once internalised, these vesicles need active transportation to get through the dense, protein rich cytosol and around cytoskeleton components towards their final destination. Long-range transport to get from the cell periphery to the cell centre runs over microtubules and is mediated by the motor proteins dynein and kinesin. Transport in the cell periphery and short-range transport inside the cell is mediated by actin and its associated motor proteins, myosins. Endosomes can be pushed forward by polymerising actin filaments forming an “actin tail” or can be transported by myosins over actin filaments. Formation of actin tails has been described in a variety of internalisation pathways. After phagocytosis, movement of phagosomes is mediated by actin tails in macrophages and *Dictyostelium*[19-21]. Also macropinosomes are propelled by an actin tail towards the cell centre [22,23]. In clathrin-mediated internalisation, actin has been implicated in several steps of the internalisation process in both mammalian and yeast cells [24-26]. However, actin requirements seem to be dependent on cell type and experimental conditions [27]. Formation of an actin tail is seen in 80% of clathrin-coated pit internalisation events but only during initial movement [10,24,25]. Small actin tails polymerise on caveolae shortly after internalisation induced by Simian Virus 40 (SV40) [28]. Actin comet tails have also been reported on endosomes and lysosomes in HeLa cells, cultured mast cells, NIH 3 T3 cell, budding yeast and *Xenopus* eggs [29-32]. Movement that is mediated by such an actin tail has no defined direction nor does it run over actin tracks. In contrast, transport mediated by myosin motors runs over actin filaments in a direction dictated by the myosin. Myosins from classes I, II, V, VI, VII, XI and X are known to play a role during one or more internalisation pathways [33]. However, these myosins are mainly associated with the first steps of internalisation being membrane remodelling and pinching off of the vesicles. So far, there are few reports on the role of myosins in trafficking of endosomes. In mouse hepatoma cells, myosin 1α (Myo1α) (an analogue of human myosin 1b) contributes to the trafficking of lysosomes along microtubules [34,35]. Myosin 6 transports recently uncoated vesicles through the cortical actin barrier after clathrin-mediated internalisation in non-polarised epithelial cells [36,37]. Myosin 5 plays a role in outbound trafficking of secretory vesicles [33].

The aim of this study was to clarify how this recently characterised pathway used by surface expressed antigens in FIPV infected monocytes, was regulated and which role is played by microtubules, actin and myosins during and after internalisation. The experiments showed that myosin light chain kinase (MLCK), myosin 1, myosin 6, microtubles and actin are involved in antibody-induced internalisation in FIPV infected monocytes.

## Materials and methods

### Viruses and antibodies

A third passage of FIPV strain 79–1146 (American Type Culture Collection (ATCC)) on CrFK cells was used [38]. FIPV strain 79–1146 is a type 2 strain which is studied extensively even though type 1 strains are predominant in the field. This is because type 2 coronaviruses are easily propagated in vitro. Polyclonal anti-FCoV antibodies were kindly provided by P. Rottier (Utrecht University, The Netherlands). The antibodies were purified and biotinylated according to manufacturers instructions (Amersham Bioscience, Buckinghamshire, UK). FITC-labelled polyclonal anti-FIPV antibodies were purchased from Veterinary Medical Research and Development (VMRD, Pullman, Washington, USA). The monoclonal antibody E22-2 recognising the N protein, was kindly provided by T. Hohdatsu (Kitasato University, Japan). The monocyte marker DH59B, recognising CD172a, was purchased from VMRD. Rabbit anti-tubulin polyclonal antibodies and monoclonal antibodies against non-muscle myosin 1 were purchased from Abcam (Cambridge, UK), rabbit polyclonal antibodies against non-muscle myosin 2a, 2b and 9b from Sigma-Aldrich (Steinheim, Germany) and goat polyclonal against MLCK and rabbit polyclonal antibodies against myosin 5a, 6, 7a and 10 from Santa Cruz Biotechnology (Santa Cruz, California, USA). Secondary antibodies and reagents: goat anti-mouse Texas Red, goat anti-mouse Alexa Fluor 350, streptavidin Texas Red, streptavidin FITC, streptavidin Alexa fluor 405, anti-rabbit Alexa Fluor 594 Zenon reagent were purchased from Molecular Probes (Molecular Probes-Invitrogen, Eugene, Oregon, USA).

### Isolation and inoculation of blood monocytes

Feline monocytes were isolated as described previously [2]. Cells were seeded on glass coverslips inserted in a 24-well dish (Nunc A/S, Roskilde, Denmark) in RPMI-1640 medium containing 10% fetal bovine serum (FBS), 0.3 mg/mL glutamine, 100 U/mL penicillin, 0.1 mg/mL streptomycin, 0.1 mg/mL kanamycin, 10 U/mL heparin, 1 mM sodium pyruvate, and 1% non-essential amino-acids 100× (GIBCO-Invitrogen, Merelbeke, Belgium). Non-adherent cells were removed by washing the dishes two times with RPMI-1640 at 2 and 24 h after seeding. The adherent cells consisted for 86 ± 7% of monocytes (as assessed by fluorescent staining with the monocyte marker DH59B). At 56 h post seeding, monocytes were inoculated with FIPV at a multiplicity of infection of 5. Between 20 and 60 cells were analysed per assay.

### Internalisation inhibition assays

Twelve hours after inoculation, monocytes seeded on glass coverslips were pre-incubated for 30 min at 37 °C with 5% CO_2_ in the presence of one of the following agents dissolved in RPMI: 20 μM Latrunculin B (ICN Biochemicals Inc., Ohio, USA), 50 μM Cytochalasin D (Sigma-Aldrich GmbH, Steinheim, Germany), 50 nM Jasplakinolide (Molecular Probes), 500 μM Colchicine (Sigma-Aldrich GmbH), 20 μM Nocodazole (Sigma-Aldrich GmbH), 5 μM Paclitaxel (Calbiochem, San Diego, California, USA), 500 nM Bisindolylmaleimide (Calbiochem), 10 μM ML-7 (Calbiochem), 500 nM H-89 (Calbiochem), 3 μM KN-93 (Calbiochem), 200 μM PKG inhibitor (Calbiochem), 150 nM K-252a (Calbiochem) and 40 μM Blebbistatin (Sigma-Aldrich GmbH). The working concentration of each reagent was based on literature values and was optimised qualitatively in internalisation assays with control ligands (data not shown). Viability of the cells during the inhibition assay was tested for each inhibitor using ethidium bromide monoazide (Molecular Probes-Invitrogen) and was always over 99%.

After pre-treatment, the cells were incubated with polyclonal biotinylated anti-FIPV antibodies in presence of one of the given inhibitors for 30 min at 37 °C. Then, cells were fixed with 1% formaldehyde, permeabilized with 0.1% Triton X-100 (Sigma-Aldrich GmbH) and incubated with streptavidin-Texas Red for 1 h at 37 °C. Next, infected cells were visualised with polyclonal anti-FIPV-FITC. The glass coverslips were mounted on microscope slides using glycerine-PBS solution (0.9:0.1, vol/vol) with 2.5% 1,4-diazabicyclo(2,2,2)octane (DABCO) (Janssen Chimica, Beerse, Belgium) and analysed with confocal microscopy. Percentages of cells with fully internalised complexes were calculated relative to the total amount of monocytes which showed antibody binding and thus had membrane expression before antibodies were added. Those monocytes constitute about 50% of the total amount of infected cells [2]. Because of the variability on the amount of cells with membrane expression, visualisation of the complexes remaining at the plasma membrane was needed. Therefore, an acid washing step to remove the extracellular antibodies was not performed.

To test the effectiveness of all reagents, a suitable control was used in each experiment. Monocytes seeded on glass coverslips were pre-incubated for 30 min at 37 °C with 5% CO_2_ in the presence of one of the inhibitors. After treatment, the cells were incubated with biotinylated transferrin (Sigma-Aldrich GmbH) or fluorescent 1 μm polystyrene microspheres, FluoSpheres (Molecular Probes-Invitrogen), in presence of the inhibitor. Then, all cells were fixed with 1% formaldehyde and permeabilized with 0.1% Triton X-100. The biotinylated transferrin was visualised by incubating the cells with streptavidin-FITC for 1 h at 37 °C and cells incubated with fluorescent beads were incubated with phalloidin-Texas Red (Molecular Probes-Invitrogen) for 1 h at 37 °C to visualise the lamellipodia. The glass coverslips were mounted on microscope slides using glycerine-DABCO and analysed by confocal microscopy. For the controls, the monocytes were scored analogously as FIPV-infected cells: ligands were considered “fully internalised” when they were only observed inside the cell. Fluorescent beads were considered internalised when they were found inside the cortical actin labelling.

### Co-localisation studies with MLCK, myosins, actin filaments or microtubules

Twelve hours after inoculation, monocytes were incubated with biotinylated anti-FIPV polyclonal antibodies. At different times post antibody addition, cells were fixed with 1% formaldehyde, permeabilized with 0.1% Triton X-100 and antigen-antibody complexes were visualised with streptavidin-FITC followed by a blocking step with 10% negative goat serum. Next, actin filaments, microtubules, MLCK or myosins were stained. Cells were incubated with phalloidin-Texas Red to visualise actin filaments. To stain the microtubules, polyclonal rabbit anti-tubulin antibodies were tagged with anti-rabbit Alexa Fluor 594 Zenon reagent. To visualise MLCK, a goat polyclonal was used, followed by rabbit anti-goat Alexa Fluor 594. To visualise myosin 1, monoclonal anti-myosin 1 was used, followed by goat anti-mouse Texas Red. To visualise other myosins, rabbit anti-myosin 2a, 2b, 5a, 6, 7a, 9b and 10 was tagged with anti-rabbit Alexa Fluor 594 Zenon reagent. After 45 min of incubation, cells were fixed to stabilise the Zenon reagent. Finally, infected cells were visualised with monoclonal anti-N and goat anti-mouse Alexa Fluor 350 (not shown in images).

For the colocalisation of myosin 1 and MLCK during the internalisation process, infected monocytes were incubated with anti-FIPV polyclonal antibodies. At different times post antibody addition, cells were fixed with 1% formaldehyde, permeabilized with 0.1% Triton X-100 and MLCK was visualised with goat anti-MLCK, biotinylated donkey anti-goat and streptavidin Alexa fluor 405 (with negative goat serum to block). Then myosin 1 was visualised with mouse anti-myosin 1 and goat anti-mouse Texas Red. The internalising antigen-antibody complexes were visualised with goat anti-cat FITC.

### Confocal laser scanning microscopy

The samples were stained as described above and examined with a Leica TCS SP2 or SPE laser scanning spectral confocal system (Leica Microsystems GmbH, Wetzlar, Germany) linked to a DM IRB inverted microscope (Leica Microsystems). Argon and Helium/Neon laser lights were used to excite FITC (488 nm line) and Texas Red (543 nm line) fluorochromes, the violet diode laser was used to excite Alexa Fluor 405 (405 nm line). The images were obtained with Leica confocal software and processed with the GIMP.

### Statistical analysis

Triplicate assays were compared using a Mann-Withney *U* test with SPSS 11.0 (SPSS Inc., Chicago, WA). *P* values < 0.05 were considered significantly different.

## Results

### Internalisation of viral antigens is regulated by myosin light chain kinase but not by myosin 2

The serine/threonine kinases are the biggest group of kinases and consist of different classes among which: protein kinase C (PKC), protein kinase A (PKA) or cyclic AMP-dependent protein kinases, protein kinase G (PKG) or cyclic GMP-dependent protein kinases, the family of the calcium/calmodulin-dependent protein kinases (CaMK) and myosin light chain kinases (MLCK). The importance of these classes was tested by performing internalisation assays in the presence of pharmacological inhibitors. PKC is known to regulate Fc receptor-, complement receptor- and mannose receptor-mediated phagocytosis [6]. PKA and MLCK were shown to mediate phagocytosis in neutrophils [39]. So for the inhibition of these targets, fluorescent beads were chosen as a control ligands. PKC is known to regulate Fc receptor-, complement receptor- and mannose receptor-mediated phagocytosis [6]. PKA and MLCK were shown to mediate phagocytosis in neutrophils [39].

**Figure 1:**
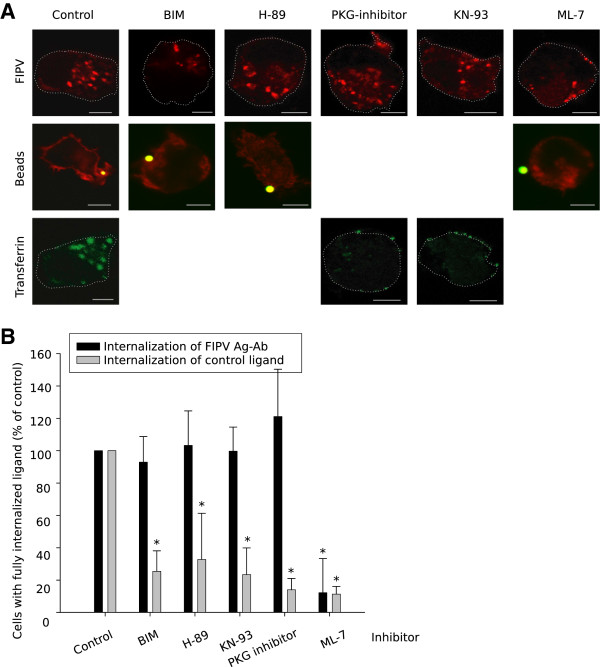
**Myosin light chain kinase inhibitors can block the internalisation of surface expressed viral antigens.** A range of serine/threonine kinases were tested for their importance during the internalisation process using chemical inhibitors: Bisindolylmaleimide (PKC inhibitor), H-89 (PKA inhibitor), PKG inhibitor, KN-93 (CaM-dependent kinase II inhibitor), ML-7 (MLCK inhibitor). **(A)** Confocal images of feline monocytes after an internalisation assays of 30 min using antibodies or control ligands: beads or transferrin. In row 2, cortical actin was stained (red) to visualise whether or not the lamellipodia were closed around the beads. The images show a single optical section through the cell, scale bar indicates 5 μm. **(B)** Quantification of the internalisation in presence of inhibitors against serine/threonine kinases. Results are given relatively to a control of untreated cells. Data are means and standard deviations of triplicate assays. The asterisk marks results that are significantly different from the untreated control (*p* < 0.05).

**Figure 2:**
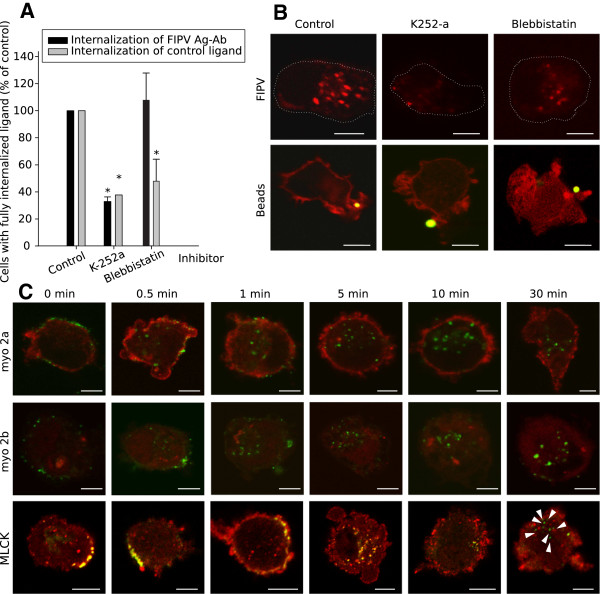
**The internalisation of antigen-antibody complexes is mediated by MLCK but not myosin 2. (A)** Quantification of the internalisation process in presence of K252-a (inhibits MLCK, PKA, PKC and PKG) and Blebbistatin (inhibits myosin 2) 30 min after addition of antibodies. Results are given relatively to a control of untreated cells. Data are means and standard deviations of triplicate assays. The asterisk marks results that are significantly different from the untreated control (*p* < 0.05). **(B)** Confocal images of monocytes after internalisation in the presence of the inhibitors. The activity of each inhibitor was tested with internalisation assays of fluorescent beads. In row 2, cortical actin was stained (red) to visualise whether or not the lamellipodia were closed around the beads. **(C)** Visualisation of myosin 2a, 2b and MLCK (red) during antibody-induced internalisation of surface expressed viral antigens (green) in FIPV-infected monocytes at some time points after antibody addition. Arrow heads in the MLCK row indicate antigen-antibody complexes were colocalisation was lost. All images show a single optical section through a monocyte, scale bar indicates 5 μm.

The internalisation of viral antigens remained unaffected in the presence of bisindolylmaleimide I (a PKC-inhibitor), H-89 (a PKA-inhibitor), PKG-inhibitor and KN-93 (a CaMK II-inhibitor) while the internalisation of a control ligand was reduced to 25 ± 13%, 33 ± 29%, 14 ± 7% and 23 ± 16% respectively (representative images and results are given in Figure 1). Additionally, it was confirmed that not the infection itself nor the presence of antibodies to induce internalisation affected the activity of inhibitors directed against the major internalisation pathways (data not shown). In contrast, the specific MLCK inhibitor ML-7, could inhibit the internalisation of viral antigens to 12 ± 21% of the untreated control and the uptake of beads, the control ligand, was equivalently reduced to 11 ± 5% (Figure 1). The importance of MLCK was reinforced by another MLCK inhibitor: K252a, which also inhibited the internalisation of both the viral antigens as the control ligand (beads) to a similar level (Figure 2A and B). It is described in literature that MLCK regulates myosin 2 both by phosphorylation of the regulatory light chain and by binding to it [40,41]. Thus, an additional internalisation inhibition assay was performed with blebbistatin, a myosin 2 inhibitor, to investigate the involvement of myosin 2. The test indicated that internalisation of the antigen-antibody complexes could not be blocked by blebbistatin, while the uptake of beads could (Figure 2A and B), indicating that myosin 2 is not involved in the internalisation process. This was further investigated with co-localisation stainings of the antigen-antibody complexes with myosin 2a and 2b and MLCK. Figure 2C shows that no indication of a role for myosin 2a nor 2b were found while MLCK clearly colocalized already before the addition of antibodies (see Figure 2C). MLCK remained associated with the viral antigen-antibody complexes at least until 10 min after antibody addition. After 30 min, the association was lost.

### Co-localisation of viral antigen-antibody complexes, myosins and MLCK

**Figure 3:**
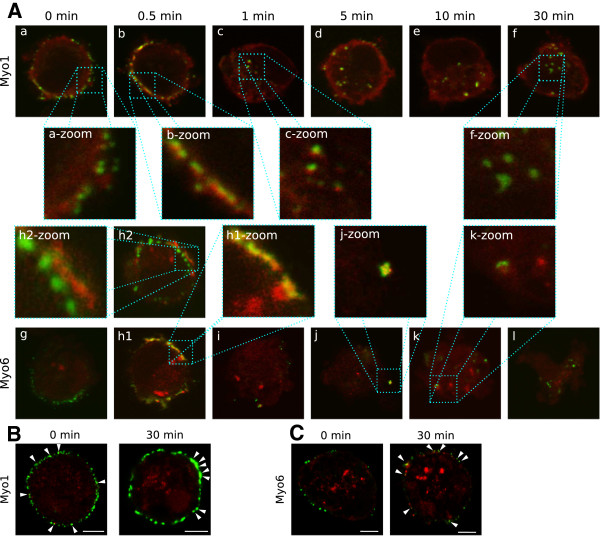
**The role of myosin 1 and 6 during the internalisation of viral antigens. (A)** Confocal images of the internalisation process at different times post antibody addition, in which the antigen-antibody complexes was visualised with FITC (green signal). Myosins were visualised in red with Texas Red (Myo1) or Alexa Fluor 594 (myo6). (B + C) The effect of ML-7 on the recruitment of myosin 1 **(B)** or myosin 6 **(C)** in the internalisation of antigen-antibody complexes. Cells were pretreated with ML-7 for 30 min. Cells were fixed before or 30 min after antibody addition. Antigen-antibody complexes were visualised in green and myosin in red. Arrowheads indicate colocalisation of myosins with antigen-antibody complexes. All images show a single optical section through the cell. Scale bars represent 5 μm.

Since the experiments suggested that myosin 2 is not involved in the internalisation process, it was further investigated which myosin is involved. Myosin 1, 5a, 6, 7a, 9b and 10 were selected based on their role during several internalisation processes. During the internalisation experiments, no co-localisation was found between myosin 5a, 7a, 9b and 10 and the viral antigens at any time point (data not shown). In contrast, viral antigens did co-localise with myosin 1 and 6. Similar to MLCK in the previous experiment, myosin 1 was highly enriched at the plasma membrane, right underneath the viral proteins (see panel a-zoom of Figure 3A) already before the antibodies were added. Then, shortly after addition of the antibodies, myosin 1 relocated between the internalised complex and the plasma membrane (see panel b-zoom of Figure 3A). As the antigen-antibody complexes moved further into the cell, they maintained their association with myosin 1. At 10 min after antibody addition, a loss of interaction was first observed (e.g. in the centre of the cell depicted in panel e of Figure 3A). Further dissociation of myosin 1 from the internalised complexes occurred as time passed and vesicles reached the centre of the cell. Co-localisation with myosin 6 was also observed as quickly as 30 s after addition of the antibodies when antigen-antibody complexes were right under the plasma membrane (see panel h1-zoom of Figure 3A) but association was lost as soon as the viral antigen-antibody complexes moved further inside the cell (illustrated in panel h2 and h2-zoom of Figure 3A). The images in panel h1 and h2 are actually different sections through the same cell, which clearly illustrates how short-lived the myosin 6 association with internalised complexes is. At later time points, cells with co-localisation were still found, but never below the actin cortex (e.g. panel j-zoom of Figure 3A). We suggest that those co-localisations represent antigen-antibody complexes that just started to internalise at the moment of fixation.

To further test whether or not MLCK could be involved in the regulation of myosin 1 or 6, it was assessed if these myosins would associate with the antigen-antibody complexes in the presence of the MLCK inhibitor ML-7. The images in Figure 3B show that if cells are treated with ML-7, the association of the antigen-antibody complexes with myosin 1 was strongly affected and occurred only in a small fraction of the complexes. At 30 min post antibody addition, a small fraction remains associated with myosin 1. However, these complexes did not internalise. When myosin 6 was stained in cells pretreated with ML-7, no association could be seen before antibody addition (similar as in untreated cells). At 30 min post antibody addition, a substantial amount of association was seen, so the call for myosin 6 seemed intact. Since ML-7 effectively inhibits internalisation, mere association with myosin 1 and/or 6 is not enough for their function.

**Figure 4:**
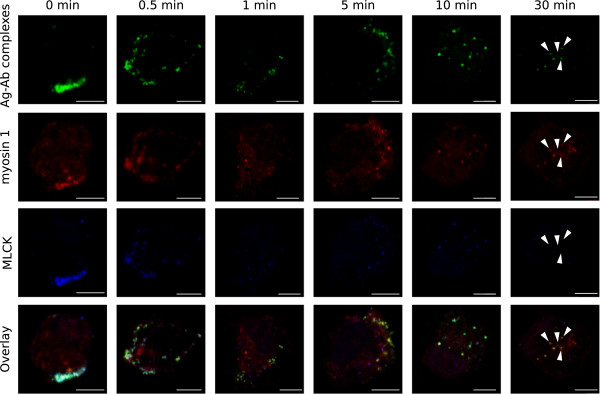
**Colocalisation staining of antigen-antibody complexes, Myosin 1 and MLCK during the internalisation process.** Confocal images of the internalisation process at different times post antibody addition, in which the antigen-antibody complexes was visualised with FITC (green signal), myosin 1 with Texas Red (red) and MLCK with Alexa Fluor 405 (blue). The images show a single optical section through a monocyte. Scale bars represent 5 μm and arrowheads indicate vesicles which have lost the association of MLCK.

Since the pattern of the myosin 1 association with the internalising antigen-antibody complexes resembles the MLCK pattern and the previous experiment indicated that recruitment of myosin 1 was most affected by the inhibition of MLCK, a co-localisation staining of the antigen-antibody complexes, myosin 1 and MLCK was done. Figure 4 confirms that the internalising complexes, myosin 1 and MLCK fully co-localize before and during the initial steps of the internalisation process. At 30 min post antibody addition, vesicles appear which still carry myosin 1, but MLCK association was lost. This is probably shortly before the association with myosin 1 is also lost, as was seen in Figure 3A. These results further confirm that MLCK might be regulating myosin 1.

### The role of actin in internalisation of viral antigen-antibody complexes

**Figure 5:**
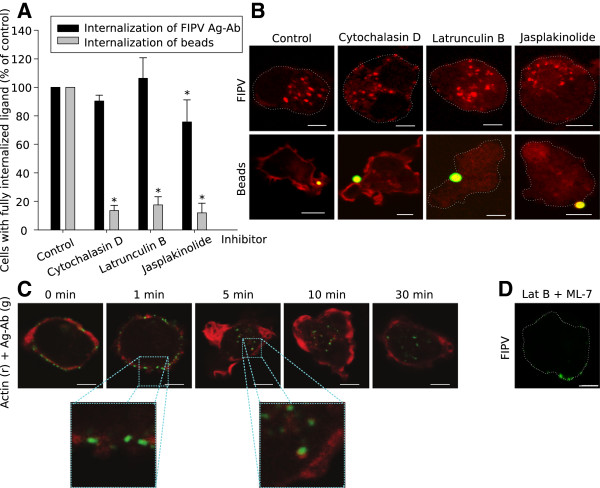
**Role of actin in the internalisation of surface expressed viral antigens in FIPV-infected monocytes. (A)** Quantification of the internalisation of surface expressed viral antigens in the presence of actin inhibitors. Results are given relatively to a control of untreated cells. Data are means and standard deviations of triplicate assays. The asterisk marks results that are significantly different from the untreated control (*p* < 0.05). **(B)** Confocal images of monocytes after internalisation in the presence of actin inhibitors. The activity of each inhibitor was tested with internalisation assays of fluorescent beads. In row 2, cortical actin was stained (red) to visualise whether or not the lamellipodia were closed around the beads. **(C)** Visualisation of actin dynamics (red) during antibody-induced internalisation of surface expressed viral antigens (green) in FIPV-infected monocytes at some time points after antibody addition. **(D)** A confocal image of an infected monocyte at 30 min post antibody addition during treatment with Latrunculin B and ML-7. All images show a single optical section through a monocyte and the scale bars represent 5 μm.

As myosins are involved in the internalization process, the tracks of these motor proteins, actin filaments, may also be involved. To investigate the role of actin during this internalisation pathway, internalisation assays were performed in presence of the inhibitors Cytochalasin D (which inhibits formation of new filaments), Latrunculin B (which disrupts all actin filaments) and Jasplakinolide (which stabilises existing filaments and induces polymerisation of new filaments). Figure 5B shows representative confocal images of monocytes at 30 min after addition of antibodies in the presence of one of the inhibitors. Cells treated with Cytochalasin D or Latrunculin B showed internalised viral antigen-antibody complexes in a pattern similar to that in the untreated control cells. Monocytes treated with Jasplakinolide also internalised viral antigen-antibody complexes, however, the typical pattern of randomly distributed internalised complexes was not observed. Fewer internalised vesicles seemed to travel as far into the cell as in untreated monocytes or in monocytes treated with Cytochalasin D or Latrunculin B. In monocytes treated with Jasplakinolide, one can expect a cortical actin network that might even be more extensive than in untreated cells because of the polymerisation inducing capacity of the drug. Nevertheless, almost no filamentous actin can be seen in the image of the monocyte trying to internalise a fluorescent bead in Figure 5B. The reason for this apparent discrepancy is that Jasplakinolide impedes phalloidin-Texas Red from binding to actin filaments resulting in a vaguely red cell even though a cortical actin network is present.

The quantification of the internalisation in the presence of inhibitors confirmed that Cytochalasin D and Latrunculin B did not have a significant effect on the internalisation process, while both inhibitors strongly reduced phagocytosis of fluorescent beads to respectively 14 ± 4% and 18 ± 6% of the untreated control (Figure 5A). These results suggest that actin filaments are not required for the internalisation process. Treatment of monocytes with Jasplakinolide gave a small but significant reduction in internalisation (76 ± 15% of the untreated control), suggesting that a stabilised cortical actin network might hamper or slow down the internalisation of antigen-antibody complexes. Since the internalisation process could not be blocked by disruption of actin filaments and internalisation itself was not stopped by stabilised filaments, it can be concluded that actin does not play an active role in the internalisation process.

To further elucidate the role for actin during the internalisation process, actin filaments were visualised with phalloidin-Texas Red at different times after initiation of the internalisation. The confocal images in Figure 5C show that at 1 min after addition of antibodies, antigen-antibody complexes moved into the cells and at the internalisation sites a local absence of cortical actin could be observed. It could be that actin filaments were moved or broken down in order to make way for the internalising complexes, which provides an explanation why Jasplakinolide reduced or slowed down the internalisation. Another noteworthy observation was made at later stages of the internalisation process. Figure 5C shows that vesicles that have passed through the cortical actin network were still associated with actin in a way that resembles actin tails. This association of internalised vesicles with actin was still observed at 10 min after initiation of the internalisation, but was lost at 30 min.

By treating the cells with a combination of Latrunculin B and ML-7, it was investigated if lifting the actin barrier would be enough to enable internalisation or if MLCK (and thus myosins) are also required to initiate the internalisation process. It was found that the internalisation in the presence of both inhibitors was reduced to 3 ± 5%, which is not significantly different from the inhibition assay with ML-7 alone, which indicate that active MLCK is required for the initiation of the internalisation process (see Figure 5D for a cell treated with Latrunculin B and ML-7 at 30 min post antibody addition).

Taking these results together, MLCK plays a role in the initiation of the internalisation process. In contrast, actin might not be required for internalisation, as indicated by the inhibition assays with Cytochalasin D and Latrunculin B. The actin stainings and the results with the actin stabilising drug Jasplakinolide indicated that the cortical actin network forms a barrier that can slow down internalisation and that must be overcome by moving or disintegrating actin filaments. Additionally, actin and MLCK may play an active role in further transportation into the cell since fully internalised complexes were associated with actin tails and MLCK.

### The role of microtubules in transportation of viral antigen-antibody complexes into the cell

**Figure 6:**
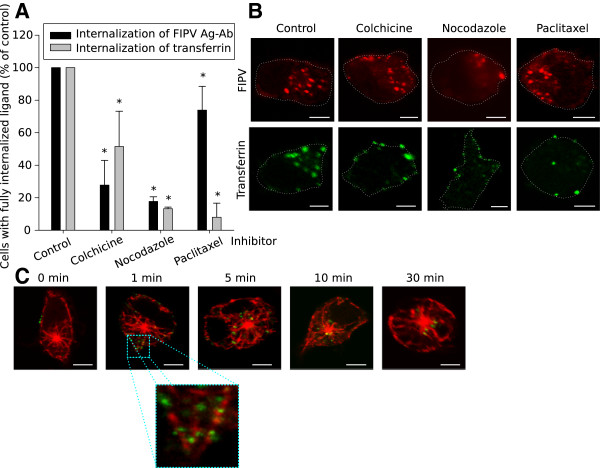
**Role of microtubules during internalisation of surface expressed viral antigens in FIPV-infected monocytes. (A)** Quantification of the internalisation in the presence of microtubule inhibitors. Results are given relatively to a control of untreated cells. Data are means and standard deviations of triplicate assays. The asterisk marks results that are significantly different from the untreated control (*p* < 0.05). **(B)** Confocal images of monocytes after internalisation in the presence of microtubule inhibitors. The activity of each inhibitor was tested with internalisation assays of transferrin. **(C)** Visualisation of the microtubules during antibody-induced internalisation of surface expressed viral antigens in FIPV-infected monocytes. All images show a single optical section through a monocyte and the scale bars represent 5 μm.

The primary route for vesicles to move from the plasma membrane towards the cell centre runs over the microtubules. The internalisation studied here is a very fast and efficient process. Internalised antigen-antibody complexes can be found in the centre of the cell as soon as 5 min after addition of antibodies. In this section we verified if internalised vesicles are transported over the microtubules to reach the cell centre. First, internalisation assays were performed in the presence of one of the following inhibitors: Colchicine, Nocodazole (which both disrupt microtubules) or Paclitaxel (which promotes the assembly and inhibits the disassembly of microtubules). The images in Figure 6B show that some internalisation could still occur in the presence of either inhibitor since antigen-antibody complexes were observed inside the monocytes. However, most internalised vesicles remained close to the plasma membrane indicating that transportation to the centre of the cell was inhibited. Quantification revealed a decrease in internalisation when monocytes were treated with Colchicine or Nocodazole, to 28 ± 15% and 18 ± 3% of the untreated control respectively (Figure 6A). Thus, not only transportation was inhibited but internalisation itself as well. Treatment with Paclitaxel also led to a decrease in internalisation, albeit less pronounced than with the other inhibitors (74 ± 15% of the untreated control). Since Paclitaxel is a drug that stabilises microtubules, these findings indicate that microtubules must not only be intact but they must also remain dynamic. Although the decrease was significantly different, it was minor compared to the strong reduction in internalisation of the control ligand transferrin (8 ± 9% of the untreated control). This indicates that the requirement for dynamic microtubules is not as stringent in the internalisation pathways studied here as it is in the clathrin-mediated internalisation of transferrin.

To confirm the role for microtubules in the transportation of internalised vesicles, microtubules were visualised during the internalisation process. The confocal images in Figure 6C show that internalising vesicles were associated with microtubules as soon as 1 min after initiation of internalisation, thus microtubule based transport started most likely right after passage through the cortical actin network. After 10 min, the first vesicles already reached the microtubule organising centre (MTOC). Association of the internalised vesicles with microtubules was maintained at all tested time points.

Taken together, these findings indicate that antigen-antibody complexes were transported over the microtubules towards the cell centre and accumulated at the MTOC.

## Discussion

When primary feline monocytes are infected with FIPV in vitro, a fraction of the expressed spike (S) protein and membrane (M) protein can be found in the plasma membrane in half of the infected cells [2]. In contrast, viral antigens were not detected in the plasma membrane of monocytes isolated form naturally infected cats [4]. When mimicking the in vivo situation by adding antibodies to the in vitro culture of FIPV-infected monocytes, we found that the S and M proteins were quickly and efficiently internalised as heterocomplexes, leaving the plasma membrane cleared from visually detectable viral antigens [3]. The internalisation of these antigen-antibody complexes occurred via a new clathrin- and caveolae-independent pathway which did not require dynamin, rafts nor rho-GTPases. Since the antigen-antibody complexes are internalised and transported towards the cell centre so rapidly, we wanted to investigate how the internalisation process was initiated and the intracellular transport was organised.

The importance of myosin motors during internalisation is best studied in phagocytosis where Myosin 1, 2, 5, 7, 9b and 10 cooperate from start to completion of the process [42-48]. The role for myosins in other internalisation processes is less well established. Myosin 1 has been associated with macropinocytosis and clathrin-mediated internalisation [49,50] and myosin 6 with clathrin-mediated internalisation [36,51]. The co-localisation stainings with myosin 1 of the internalisation process studied here, suggest that this myosin might be the driving force behind the membrane invagination. It has been described that Myosin 1E (formerly known as 1C) might indeed couple polymerizing actin to membranes and thus mediate force production during endocytosis through constraining (and possibly orienting) actin assembly [52,53]. Several studies mention that myosin 1 is involved in the formation of internalising vesicles [54,55]. We found that patches of myosin 1 could be observed under the viral proteins residing in the plasma membrane. It could be that the binding of antibodies on the viral proteins induces a conformational change that exposes a signal sequence in the cytoplasmatic tail of the protein leading to the activation of the internalisation machinery.

The co-localisation stainings also indicated a role for myosin 6 in the first steps of the internalisation process. In non-polarised epithelial cells, the short isoform of myosin 6 transports recently uncoated vesicles through the actin barrier [36,37]. If myosin motors are coupled as dimers, they can mediate directed movement over a filamentous actin network such as cortical actin [56-58]. In the study presented here, we used primary monocytes, which are non-polarised cells containing a cortical actin network. It is highly probable that myosin 6 will perform the same task here, namely transporting the internalised antigen-antibody complexes through the cortical actin.

The results further show that MLCK is required for the internalisation process, as are myosin 1 and 6 but not myosin 2a nor 2b. In literature it is stated that MLCK has a substrate specificity restricted to the regulatory light chain of myosin 2. However, most research has been done on muscle cells while MLCKs have been shown to function differently in non-muscle cells [59]. So, it is possible that there are members of the MLCK family that are not restricted to myosin 2. Research by Blue and colleagues has indeed shown that the 220 kDa non-muscle MLCK isoform does not co-localise with myosin 2a nor 2b, while it did co-localise with actin filaments [60]. Our results hint towards the existence of an MLCK isoform that regulates myosin 1. This is suggested by (1) the co-localisation kinetics in which myosin 1 co-localises with the visualised MLCK isoform before and during the internalisation process, (2) the finding that recruitment of myosin 1 to the antigen-antibody complexes is hampered by treatment with ML-7. This inhibitor blocks the catalytic activity of MLCK, preventing the phosphorylation of its substrate: myosin [61], and (3) the finding that myosin 1 and MLCK are already associated with the viral antigens before addition of antibodies and thus before internalisation, indicates that mere binding of MLCK is not enough but its kinase activity is of importance.

When the role for actin was investigated, it was found that in the internalisation pathway studied here, dynamic actin was not a prerequisite. This hypothesis is further corroborated by the co-localisation stainings which clearly show that the cortical actin network forms a barrier that must be moved aside or locally degraded to allow the internalising vesicle to pass through. Similar observations have been made during clathrin-or caveolae-mediated internalisation [27,37,62]. In the internalisation process that was studied here, myosin 1 and/or 6 could play a role in moving the cortical actin, but this is not the only role for (at least) myosin 1 since taking away the cortical actin in an ML-7 treated, thus MLCK inactive, cell did not enable internalisation. This, combined with the finding that myosin 1 is already associated with the antigens before antibody binding, strongly suggests that myosin 1 is required for the initial steps of the internalisation process, e.g. membrane remodelling.

**Figure 7:**
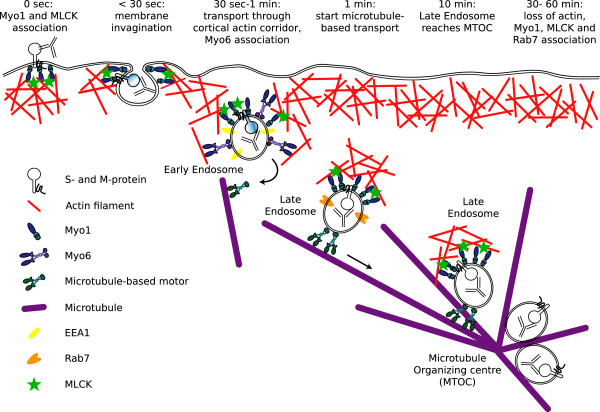
**Model of the clathrin- and caveolae independent internalisation pathway used for antibody-induced internalisation of viral antigens in FIPV-infected monocytes.** In this figure, the data obtained in this publication and previous work has been compiled in to one model of the internalisation pathway followed by the antigen-antibody complexes upon binding of specific antibodies.

Once passed through, the internalised vesicles are further transported over microtubules. The track switch from actin to microtubules might be mediated by myosin 6 [63]. As soon as the vesicles move over the microtubules, association with myosin 6 was lost while association with myosin 1 was maintained. It could be that myosin 1 and actin filaments cooperate with microtubules during intracellular trafficking. Similar observations have been made in mouse hepatoma cells where myosin 1α (an analogue of human myosin 1b) contributes to the trafficking of lysosomes along microtubules by controlling the directionality of the long-range movements [35]. Evidence is accumulating for the existence of motor complexes that combine actin- and microtubule-based transport although efforts are focused on Myosin 5-mediated outbound trafficking [63,64]. Studies also indicate that myosin 1 controls and constrains actin polymerisation rather than promoting nucleation [52,65,66]. The experiments indeed showed that intracellular transport did not require dynamic or polymerising actin. So, it is possible that the actin tails on the internalising vesicles are not required for propelling the vesicle but merely to help in orienting its trafficking towards the cell centre under the control of myosin 1 which constrains the actin polymerisation. A hypothetical model combining all results on the internalisation process, with indication of endosome markers as was found in previous work, is given in Figure 7[18].

From this research, an interesting target for a new therapy against FIPV arises: MLCK. By inhibiting MLCK with ML-7, the internalisation process could be efficiently blocked, which enables the immune system of the host to recognise and eliminate the infected cells. Such an anti-immune evasion therapy is a whole new approach to treat chronic infectious diseases and has some important advantages over classical anti-viral therapy: (1) A cellular target is used, hence no problems of drug resistance and (2) the treatment might be useable for other immune-evading viruses; (3) It might lead to elimination of the virus since the virus pools can now be targeted.

In conclusion, the clathrin- and caveolae-independent internalisation pathway through which surface expressed viral proteins are internalised after antibody binding in FIPV-infected monocytes, was initiated and driven by myosin 1 and MLCK, but did not require actin. The experiments indicate that myosin 1 might be the driving force of the internalisation process after activation (phosphorylation) by MLCK. During passage through the cortical actin network, myosin 6 associated with the antigen-antibody complexes as well. Once passed the cortical actin, microtubule-based transport started and association with myosin 6 was lost. During transport over microtubules, the vesicles were associated with small actin tails, MLCK and Myosin 1, indicating that actin and microtubules cooperate during intracellular trafficking, probably mediated by Myosin 1. After 10 min, the internalised vesicles reached the microtubule organising centre where they accumulated and the actin tails, MLCK and myosin 1 associations were lost from 30 min on.

## Competing interests

HLD and HJN are currently applying for a patent on MLCK as a therapeutic target.

## Authors’ contributions

HLD and HJN conceived the study. HLD designed and performed the experiments, analysed the data and wrote the manuscript. YN and LMD performed the experiments with the inhibitors. All authors read and approved the final manuscript.
